# Computational perspectives on tubulin E-hook structure and mechanisms

**DOI:** 10.1016/j.bpr.2026.100249

**Published:** 2026-01-08

**Authors:** Alexander C. Bromley, Dana N. Reinemann

**Affiliations:** 1 Department of Biomedical Engineering, University of Mississippi, University, Mississippi 38677; 2 Department of Chemical Engineering, University of Mississippi, University, Mississippi 38677

## Abstract

E-hooks, or the C-terminal tails of tubulin, mediate interactions between microtubules and associated proteins. Despite their functional importance in cellular and physiological processes, their structural variability and mechanistic roles remain poorly understood. E-hooks are thought to be intrinsically disordered to some degree, making crystallographic studies difficult and necessitating the use of computational tools to study their structures and how they change E-hook function. This review synthesizes recent computational efforts to elucidate E-hook structure, dynamics, and functional differentiation across tubulin isotypes. We examine studies probing E-hooks in isolation or with globular cores, which have revealed subunit-specific features influencing microtubule behavior. We also evaluate the role of E-hooks in modulating binding affinity and conformational states of motor proteins and microtubule-associated proteins. Finally, we highlight adjacent technological and methodological advances that have implications for both the interpretation of past findings and the design of future studies, offering new directions for the investigation of E-hook-mediated microtubule regulation.

## Why It Matters

Microtubules, essential cellular highways, are coated with E-hooks, a brush of flexible, electronegative tails on α- and β-tubulin that extend from the microtubule surface. These tails control how motor proteins and other binding partners interact with microtubules. Their structure makes them difficult to study experimentally, but computational modeling captures their rapid movements and electrostatic interactions. Simulations reveal how subtle sequence differences and chemical modifications fine-tune microtubule stability and protein recognition. By uncovering the molecular principles of the “tubulin code,” these studies explain how microtubule E-hooks aid in regulating essential cellular processes such as transport and organization.

## Introduction

Microtubules (MTs) provide an essential structural framework for the cell, act as cellular highways, and participate in key dynamic cell processes, such as mitosis (1), motility (2), and signaling (3). MTs are composed of αβ tubulin heterodimers (4,5). α-Tubulin and β-tubulin are each composed of a conserved, globular core and a carboxy-terminal tail that is highly disordered and varied (6). The carboxy-terminal tails are also known as E-hooks due to the high number of glutamate residues that they contain (7). These heterodimers polymerize longitudinally, forming long protofilaments that then come together and polymerize laterally to form the hollow cylinder that is a MT (8). Following this formation, the MT can continue to polymerize at the plus end, which is adorned with GTP-bound heterodimers, forming a GTP-cap (9,10). If at any point GTP hydrolysis outpaces the binding of GTP-bound heterodimers and the GTP-cap falls below a certain density threshold, then a catastrophe event will occur causing depolymerization of the MT at the plus end (9,11). The GTP-cap density, however, can be restored above the density threshold, known as a rescue event, causing polymerization to occur once more (12). This ability to rapidly grow and shrink according to needed function is known as dynamic instability (11).

Although dynamic instability is focused primarily on the core of the heterodimer, the E-hooks of the tubulin subunits serve as sites for customization and modification to meet different functional requirements (13,14). E-hooks have a variety of isoforms for each subunit, which are adjusted according to the function and location of the E-hook (15,16,17,18). These seemingly slight differences have been shown to have drastic effects on the motor proteins and MT-associated proteins (MAPs) that E-hooks bind (19,20,21,22). This function as a specialized binding site also makes E-hooks prime targets for regulation, giving the cell further control over specific MT interactions and dynamics. This regulation comes in the form of posttranslational modifications (PTMs) such as glycylation (23), glutamylation (24), phosphorylation (25,26), and more. The structural uniqueness of the isotypes integrated with these regulatory systems is known as the tubulin code (7,27,28). Briefly, the tubulin code postulates that the specific modifications made through isotype expression and PTMs modulate the functionality of MTs by affecting structural order or by changing the recruitment and binding of MAPs (27,29).

The regulation provided by the tubulin code is necessary as deviations can disrupt key mechanisms involved in fundamental cellular processes that scale to organism-level functions. E-hooks and their isotypes play specific roles in cellular organization (30), chromosome movement (30), mitotic spindle formation (31), cellular motility (32), and cellular transport (33). When this regulatory system is perturbed through isotype upregulation, aberrant PTMs, or altered MAP interactions, cytoskeletal organization and cellular function are compromised. For example, excessive tyrosination of the α-tubulin E-hooks within neurons is associated with Purkinje cell degeneration and eventual death within developing rats (34). On the other hand, reduced tyrosination of dendrites is associated with degradation of the synaptic space and is one of the steps toward Alzheimer's disease (35). Overexpression of the βIII isotype has been speculated to be linked to multiple cancers; however, it is thought to be a consequence of the cancer rather than a cause (36). Together, these alterations highlight how sensitive E-hook-MAP interactions are to changes in the tubulin code and how such disruptions can cascade into cellular dysfunction.

Understanding how changes in E-hook structure and expression influence binding behavior requires consideration of their fundamental structural features. Experimental determination of E-hook structures by x-ray crystallography has proven unsuccessful due to the peptide’s intrinsically disordered nature (37). Consequently, progress toward elucidating the mechanisms underlying E-hook function has been limited within the scope of experimental studies.

## Computational investigations of E-hooks

Computational studies addressing the limitations of experimental approaches primarily consist of molecular dynamics (MD) simulations (38,39). These simulations employ classical mechanics to simulate the motions of atoms under the influence of interatomic forces defined by a force field, providing both a visual aid and a time-resolved snapshot of the system that can be analyzed mathematically (40). A force field consists of a set of equations and parameters describing bonded and nonbonded potential energy terms, from which the forces on each atom are derived (41). These parameters, obtained through experimental means or quantum mechanical calculations, define the accuracy and behavior of each force field (41). MD simulations are commonly used to probe system-specific dynamics such as binding energetics, conformational rearrangements, and diffusion behavior and can be adapted through specialized techniques to suit the system of interest (Table 1).

**Table 1:** Computational Methods for Investigating E-hooks

Category	Calculation Method	System Description	Applications
Model resolution	All-atom molecular dynamics (MD)	Explicit representation of all atoms using a classical force field	Conformational sampling (42), ligand binding (43)

	Coarse-grained simulation	Combines multiple atoms into a singular unit/force field	Large systems (44), lipid membranes (45), protein conformation (46,47)

	Quantum mechanics calculations	Calculates forces from the electronic structure instead of classical mechanics	Higher resolution structures (48,49), chemical reactions within a simulation (50)

Techniques	Steered MD	Adds a tunable force to a selected region of the calculation	Kinesin detachment (51), MT mechanical properties (52)

	Nonequilibrium MD	Applies external field to system to observe response	Active motor impact on polymers (53), effect of electromagnetic fields (54)

	Accelerated MD	Reduces steep energy barriers while leaving others unaffected	Exploring motor protein conformational shifts during walk cycle (55,56)

Comparison of molecular simulation methods, system representations, and example applications.

Coarse-grained models combine different atoms into a single unit, providing a simplified force field for the entire unit rather than a field for each atom within it (41). These models allow for simulating larger systems (44) by decreasing computational cost and provide insights into the importance of different groups within the system at the cost of atomistic resolution (46,57). To minimize this loss of detail, the force fields must be reparametrized with additional scrutiny paid to the new coarse grains. Coarse-grain simulations have been popularized studying lipids and protein conformation with the usage of force fields such as pSPICA (45) and UNRES (47), respectively.

Different simulation techniques can also be utilized to learn targeted characteristics about the system or see how a system responds to various environments. One such protocol is guiding the motion of molecules within the simulation. Steered molecular dynamics (SMD) simulations return to the atomistic scale but apply tunable forces throughout the lifetime of the calculation (58). SMD has been used to study protein denaturing (59) as well as kinesin’s detachment from the MT (51). Other techniques utilized in MD can be found in Table 1.

Another computational technique used to investigate E-hook structure is quantum mechanical (QM) calculation (49). QM calculations utilize quantum mechanics to solve for the electronic system of the molecule targeted as opposed to expressing each atom as a unique, unchanging force field (60). The exact equations used during the calculations are determined by the chosen method. These methods tend to be density functional theory, such as B3LYP (61), or wave function approximations, such as Hartree-Fock (62), due to the multielectron nature of biomolecules. Additionally, the terms used to approximate the electron system can be changed by altering the basis set, allowing for the addition of additional Gaussian and diffuse functions (63). This significantly increases the accuracy and resolution of the atoms calculated using QM, especially in regard to intermolecular and intramolecular forces, at a significant increase in computational cost (64). When used in combination with MD, QM is typically reserved for areas that are subject to chemical reactions, an area that MD cannot model accurately; thus, the cost increase is kept to a minimum. It is possible, however, to perform significantly smaller scale simulations using QM calculations alone.

E-hook computational studies have generally followed a common workflow. First, the tubulin heterodimer is obtained from one of several protein databanks. The E-hooks are then constructed separately due to their absence in crystallographic studies. The constructed E-hook is then resolved and attached to the tubulin heterodimer. However, in some cases, the E-hook alone may be studied to examine binding interactions or to reduce computational cost. After parameterization, this structure is ready to be studied with the appropriate computational tools.

### Structural studies of β-tubulin E-hooks

As mentioned previously, both α- and β-tubulin E-hooks are widely considered to be intrinsically disordered, as well as highly negatively charged; however, the two play different roles within the MT (65,66). β-Tubulin E-hooks are much longer, sitting at 18–24 amino acids compared with α-tubulin’s 9–11 (7) (Table 2). This allows β-tubulin E-hooks to extend further from the globular core and reach out into the cytoplasm, acting as a negative brush to capture and guide potential MAPs.

**Table 2:** E-Hook Sequences

Sequences of human α- and β-tubulin E-hooks at pH 7 (67). Negatively charged residues are in red with a “-” superscript, and positively charged residues are in blue with a “+” superscript. Although histidine is only partially protonated at pH 7, its hydrogen-bonding capacity and the highly acidic local environment are expected to increase its effective pKa. Sequences were obtained from the NCBI protein database (68).

**Figure 1:**
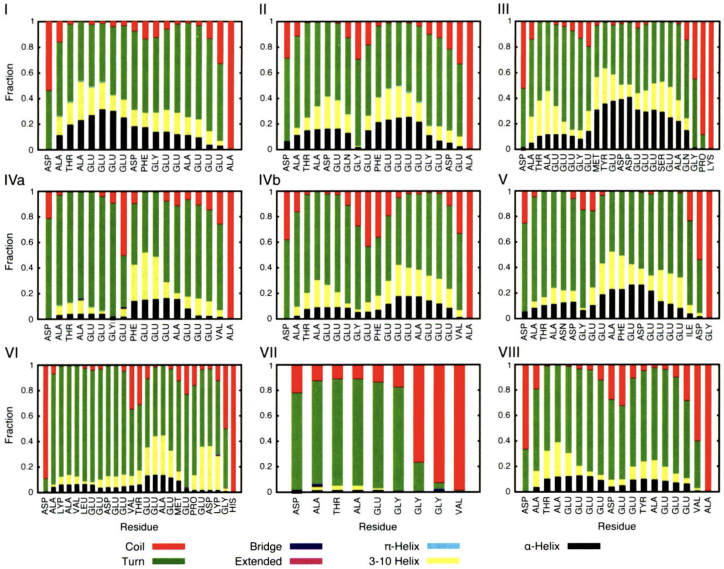
E-hook transient secondary structure. E-hooks spend most of their time in a transitive secondary structure. Each graph below shows the fraction of time each residue of a β E-hook isoform (*listed by number at the top left of each chart*) spends in various secondary structures (42). Secondary structures are color coded in the figure. Adapted from *Biophysical Journal*, Luchko et al., Conformational analysis of the carboxy-terminal tails of human β-tubulin isotypes (2008) (42). Used with permission under license number 6020610912211.

The first structural study to suggest this brush-like behavior modeled a tubulin subunit with an attached β-C-terminal tail (CTT) isoform in water to examine the conformational stability and flexibility of β-tubulin E-hooks (42). This simulation was performed individually for β isoforms 1–8. Throughout most β isoforms tested, several different helical conformations were almost equal in representation, supporting the intrinsically disordered designation (Fig. 1) (42). Although the overall conformation would frequently change, a coiled secondary structure, dependent on only a few bond angles, would consistently form, dissolve, and then reform. The E-hooks also demonstrated a great deal of flexibility throughout the isoforms, lending credence to the MAP searching function (42). However, the constant reformation of the same secondary structure may suggest a conserved secondary structure functionality, an idea previously unknown to E-hooks.

**Figure 2:**
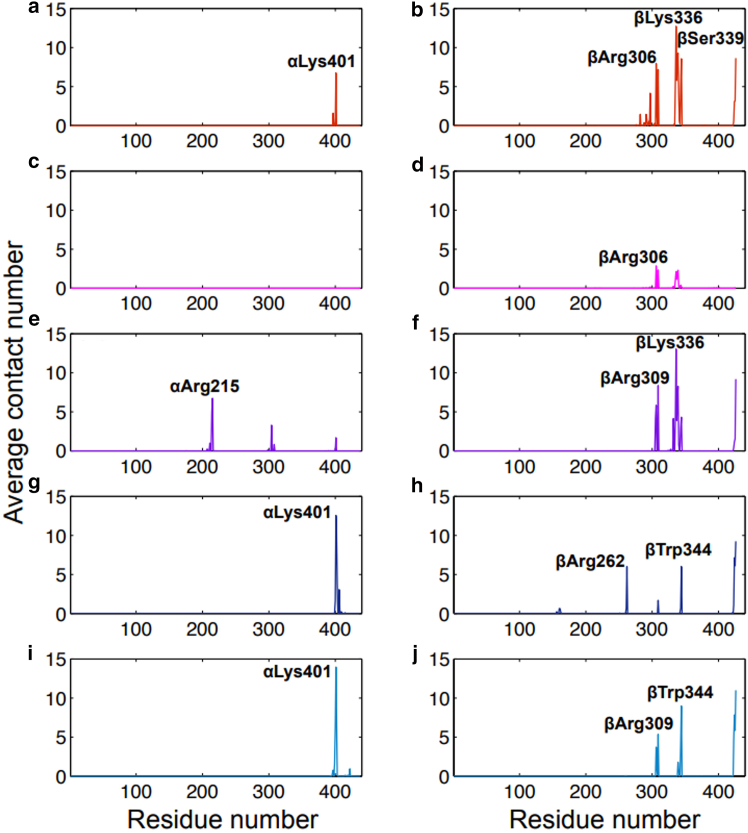
E-hook contact points with tubulin core. Residues within the β-tubulin E-hook of the αI/βI isotype system that contact with tubulin’s surface and acted as an anchor point (69). Left panels (*a*, *c*, *e*, *g*, and *i*) show contacts with the α-tubulin subunit, and right panels (*b*, *d*, *f*, *h*, and *j*) show contacts with β-tubulin subunits. Colors represent five MD trajectories (red, *a*-*b*; magenta, *c*-*d*; purple, *e*-*f*; dark blue, *g*-*h*; light blue, *i*-*j*). Reprinted (adapted) with permission from Laurin, Y., Eyer, J., Robert, C. H., Prevost, C. & Sacquin-Mora, S. Mobility and core-protein binding patterns of disordered C-terminal tails in β-tubulin isotypes. *Biochemistry* 56, 1746-1756 (2017). (69) Copyright 2025, American Chemical Society.

In another MD study, researchers used an atypical OPLS-AA (Optimized Potentials for Liquid Simulations-All Atom) force field. The protocol involved initial in vacuo minimization, followed by introduction into an aqueous environment and charge neutralization, with simulations run for a total of 100 ns quintuplicate. E-hooks were found to have secondary attachment points with the globular core (69). These attachment points, defined as a heavy atom (oxygen or nitrogen) coming within 4 Å of the globular core, were not always the same throughout different simulations but remained stable once established. Importantly, this could be due to the chosen simulation length of 100 ns as suggested by later studies (70). Fig. 2 shows these attachment points for the αI/βI system with the number of contacts each globular core residue makes averaged over the entire simulation plotted against the core residue numbers (69). Panels on the left (a, c, e, g, and i) and panels on the right (b, d, f, h, and j) show the α- and β-tubulin core contacts, respectively, and each row (e.g., panels a and b, c and d, etc.) is a replicate simulation of the same system (69). Due to the high number of negative charges along the E-hook, it preferentially attaches to residues with positive charges or dipoles. As such, although the contact points can and do change between simulations, the residues acting as contact points are primarily lysines and arginines. However, due to their relatively low concentration within the tubulin core and the E-hook conformational space being limited by its size, repeat attachment points can be seen: αLys401 in panels a, g, and i; βLys336 in panels b and d; and βArg309 in panels f and j as examples (69). Nearly all of these contact points lie within or near the interface between the α- and β-tubulin subunits, most likely due to the βI’s E-hook having a relatively short composition. In contrast, another αI/βIII system simulated showed a propensity for βIII E-hooks to make attachment points farther away from this interface, potentially due to its longer length and terminal lysine residue allowing it to make interactions that are impossible for purely negative E-hook isotypes (69). Additionally, these limits on available attachment residues and length of the β-tubulin E-hooks prevented a completely flat conformation. As such, their conformational space was limited by this additional bond, but they were still able to act as an electronegative brush for the MT (69).

**Figure 3:**
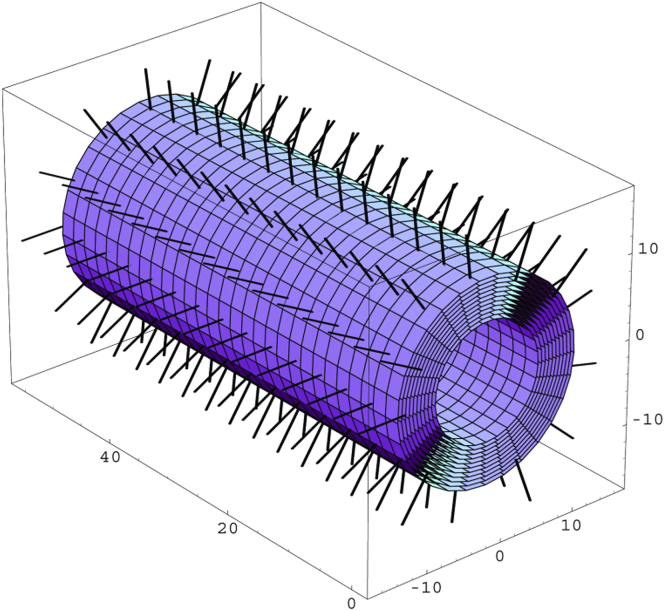
β-Tubulin E-hooks occupy a statistically upright position. E-hooks shown as thermal cones laid orthogonally against the microtubule core to reduce repulsion from nearby negative charges (71). Adapted from *Neuroquantology*, Georgiev et al., Conformational dynamics and thermal cones of C-terminal tubulin tails in neuronal microtubules (2007). Reused under Creative Commons Attribution-Noncommercial 4.0 International License.

Energetic calculations simulating E-hooks as thermal cones have shown that the upright position that β-tubulin E-hooks occupy are far more statistically likely to occur than a “down” position in which the E-hook is closer or flat against the MT core (71). Being mostly in the upright position minimizes the amount of time that β-tubulin E-hooks can remain in a position that compromises their ability to guide MAPs onto their respective microtubule-binding domain (MTBD) (Fig. 3) (71). Another computational MD study conducted conformational sampling among β-tubulin E-hooks; however, they performed their search using αI/βI- and αI/βIII-tubulin systems alone and attached to tubulin core (72). E-hooks attached to the core showed a much smaller set of conformational minima, suggesting that the tubulin core itself greatly limits the conformational sample space even without anchoring (72). This is likely due to the repulsion from negative charges along the surface of the MT and earlier residues being sterically inhibited from forming interactions with the rest of the folded E-hook.

### Structural studies of α-tubulin E-hooks

Many properties of β-tubulin E-hooks contrast sharply with those of α-tubulin E-hooks. α-Tubulin E-hook’s smaller size prevents it from acting as a general negative brush-like β-tubulin E-hooks. As such, their proposed main functionality is to regulate the MT globular core with several experimental and computational studies supporting this theory.

**Figure 4:**
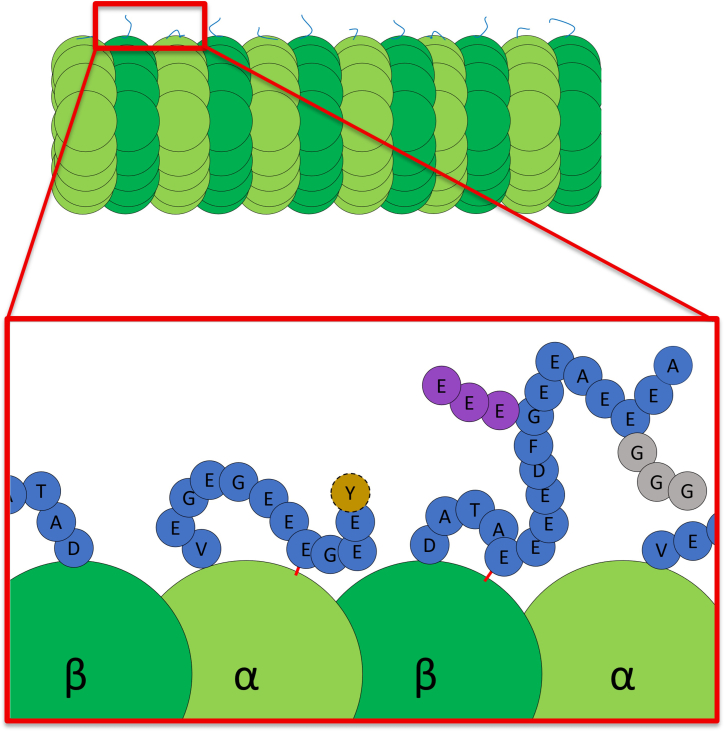
E-hook anchoring points and posttranslational modifications. Cartoon representation (*not to scale*) of a microtubule with a β-tubulin E-hook and α-tubulin E-hook with anchoring points (*red lines*). Globular core subunits are represented in green with their appropriate Greek lettering. E-hook sequences are represented in blue using their one letter coding to distinguish specific residues. Polyglutamylation, polyalkylation, and detyrosination are shown in purple, gray, and yellow, respectively.

α-Tubulin E-hooks, similar to β-tubulin E-hooks, are secondarily anchored to the core of the MT by the heavy atoms in arginine and lysine; however, these anchor points force the α-tubulin E-hooks to adopt flatter conformations, prioritizing interactions with subunits rather than the cytoplasmic environment (70). These flatter conformations can be reversed, however, with the introduction of a MT lattice expander, allowing α-tubulin to interact with MAPs (70). These lattice expanders, mainly Taxol and several MAPs, elongate the longitudinal filaments of tubulin, providing space in between the heterodimers (70). Notably, this study utilized 300-ns-long simulations, showing that anchor points can break throughout the course of the simulation (70), further opening the α-tubulin E-hooks for interactions. Additional support for this theory was found within a computational study investigating the conformational effects of PTMs. Using both all-atom and coarse-grained MD simulations of the tubulin dimer and MT lattice, the α-tubulin E-hooks were shown to have, on average, more interactions between the E-hook and the globular core than β-tubulin E-hooks, reducing the conformational flexibility available to α-tubulin E-hooks before any PTMs were applied in the simulation (73) (Fig. 4). Although these structural studies potentially point toward compositional differences in α-tubulin E-hooks leading to more core binding, their visualizations show other factors potentially at play. α-Tubulin E-hooks’ size could limit their effective space search, leading key residues to the core more often. Additionally, α-tubulin E-hooks can reach the negative end of the heterodimer, making the local environment more prone to electrostatic interactions than β-tubulin E-hooks, which are stuck in the middle of the heterodimer.

Additional corroboration was discovered when α-tubulin E-hooks and their posttranslational modifications were investigated both experimentally and computationally at the plus end of MT polymers (74,75,76). The idea that the α-tubulin E-hook interacts with the MT subunits and affects dynamic instability directly was reinforced when it was discovered that it could inhibit polymerization by transiently blocking a polymerization interface at the plus end of the MT, in agreement with previous sentiment; however, it was also discovered that tyrosinated α-tubulin E-hooks selectively bind polymerization promoters (EB1 (74) and CLIP-170 (75)). The presence and binding of both promoters at once produced a synergistic effect, increasing polymerization rates significantly. The binding of CLIP-170 alone, however, did not increase polymerization rates but did increase the rescue rate of tyrosinated MTs (76).

### Alternative structural studies

**Figure 5:**
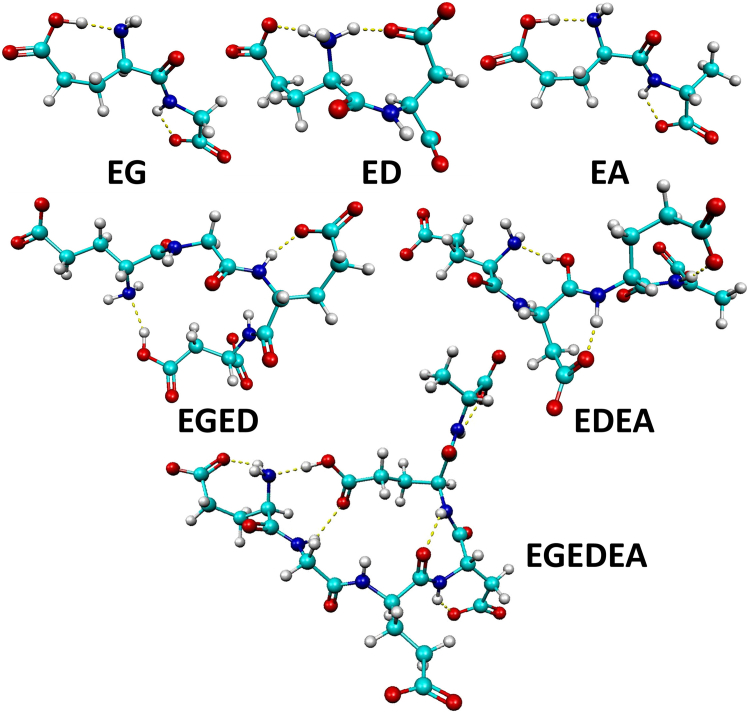
Quantum mechanical structure buildup approach. Optimized geometries for the β-II dimers, tetramers, and hexamer calculated at the B3LYP/6-311+G(2*df*,2*pd*) level with intramolecular hydrogen bonds represented by a dotted line (49). Reprinted from *Spectrochimica Acta Part A: Molecular and Biomolecular Spectroscopy*, Williams, A. et al., Determination of vibrational positions in the E-hook of β-tubulin, 118895, 2021, with permission from Elsevier.

MD simulations have been useful in detailing the dynamic properties of E-hooks, such as their interactions with their binding partners and their conformational changes. However, the small size of E-hooks, their intrinsically disordered nature, and their rapidly fluctuating structure make it challenging for MD simulations to capture a single conformation’s secondary structure and the interactions that govern it. Quantum mechanics, on the other hand, has previously been applied to study smaller peptides and their conformations (77,78,79), using Raman spectroscopy as an experimental corroboration to validate the predicted structure (80,81). In a 2021 study, these methods were applied to E-hooks using the Gaussian software (49). Due to the increased computational cost of quantum mechanics, the final C-terminal six amino acids of the β-II E-hook were taken as a representative peptide (Fig. 5). β-II was chosen due to its EGEDEA composition being highly electronegative and representative of E-hooks as a whole and due to previous QM studies calculating structures of smaller peptides of these constituent amino acids corroborated by experimental Raman spectroscopy (49). To reduce the computational cost of QM calculations and the large conformational space of the hexamer, a stepwise buildup approach was utilized. The representative hexamer was broken down into dimers, and each dimer optimized using progressively larger basis sets, concluding at the triple-ζ level. This level includes sufficient Slater-type orbitals, diffuse functions, and polarization functions to accurately describe these anionic systems. By limiting the conformational search at each stage, this approach enabled efficient convergence of otherwise expensive calculations (49). These dimers were then joined within a modeling software to elongate the protein from the C-terminus into a tetramer and optimized again (49). Repeating the process once more results in the fully optimized hexamer.

**Figure 6:**
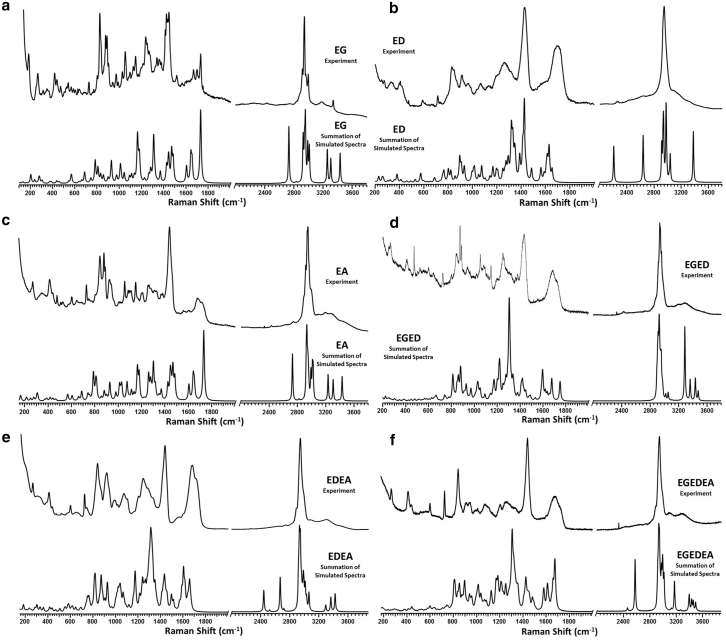
QM structure corroboration by experimental Raman spectroscopy. Experimental Raman spectra compared with their respective Boltzmann-summed simulated Raman spectrum for (*A*) EG, (*B*) ED, (*C*) EA, (*D*) EDED, (*E*) EDEA, and (*F*) EGEDEA peptide fragments in wavenumbers, cm^-1^ (48). The close agreement between experimental and simulated spectra validates the computational model employed. Reprinted from *Molecules*, Williams et al., Tracking the Amide I and alphaCOO^-^ Terminal v(C=O) raman bands in a family of L-Glutamic acid-containing peptide fragments: a raman and DFT study (2021), Licensed under CC BY 4.0.

Theoretical Raman spectra were obtained for each optimized geometry and compared with experimental spectra obtained from synthetic E-hook peptides (Fig. 6). Strong agreement between the theoretical and experimental spectra suggests that accurate E-hook structures and populations of conformations can be generated using this structural buildup approach (49). Further analysis of the optimized structures revealed the early formation of coiled and α-helical secondary structures previously observed in MD studies and indicated that higher-order structures could potentially form when MAP binding sites stabilize interactions (48). These results demonstrate that individual E-hook conformations can be studied at QM resolution, providing detailed insight into the residues and mechanisms that give rise to these structures. Although this approach does not capture the full conformational landscape of E-hooks, it complements MD studies by offering higher-resolution snapshots that clarify the structural roles of specific amino acids across varying isotype compositions (42).

## Motor protein interactions with E-hooks

Kinesin is a motor protein integral in cellular functions such as motility (82), mitosis (1), and transport (83), as well as broader physiological processes like nerve function (84). Kinesin structural and mechanochemical properties have been reviewed extensively elsewhere (85,86,87,88,89); here, features most relevant to E-hook interactions are summarized. Kinesins are a superfamily of motor proteins with 14 subsets classified according to cellular functions, overall structure, and where the motor is expressed. The position of the motor domain determines its directionality along MTs, where motor domains at the N-terminus move toward the plus end, motor domains at the C-terminus move toward the minus end, and those with centrally located motor domains often act as depolymerizers (90). Despite functional diversity, the motor domain is highly conserved, including the MT binding domain, with the primary differences occurring in either loops between the common elements of the domain or within the neck-linker portion (91).

Kinesin-1 is one of the most studied kinesins due to its high processivity and role in cargo transport. Kinesin-3, on the other hand, has been widely used in computational studies regarding E-hooks due to its super-processivity (92). This super-processivity is tied to kinesin-3’s unique K-loop, which features multiple positive lysine residues, increasing the electrostatic interactions between kinesin-3 and the MT E-hooks (93). Removal of either the K-loop or the E-hooks significantly decreases the processivity of KIF1A (94). Interestingly, transplantation of the KIF1A K-loop into kinesin-1 does not provide the same super-processivity, suggesting slight differences in binding between kinesin classes or that stepping modes are significantly affected by the divergence in these loops (93,94). Reinforcing a kinesin’s K-loop (or respective region) with additional positive charge or increasing the negative charge available on the E-hooks increases the processivity of these kinesins, bolstering the idea that the main interaction between kinesins and E-hooks is electrostatic in nature (93,94,95). Additional studies have shown that kinesins have class-specific interactions with different E-hook isotypes and PTMs. For example, an αIa/βII MT system significantly reduces kinesin-2’s velocity and processivity compared with porcine brain MTs, but kinesin-1 is largely unaffected by the change (21).

Although electrostatic interactions have been suggested to largely mediate kinesin recruitment and binding, the structural context of the E-hook-kinesin interface likely drives distinct functional behaviors observed among kinesin classes. Computational studies have been carried out to elucidate the molecular basis of kinesin-E-hook interactions. The first of these studies examined how E-hooks influence kinesin-MT binding using a coarse-grained simulation approach (96). By inducing a strong electrostatic force, the E-hooks were found to provide soft, guided landings for kinesins (96). Once near the MT binding domain, however, the E-hook would slightly repel the motor protein, decelerating the interaction speed (96). Similar results were found for dynein’s MTBD using an all-atom MD simulation (97). Contacts between E-hooks and the MTDB were found to be dynamical, and multiple negatively charged patches of amino acids on the E-hooks grab and release the same positively charged patches on the MTBD as it approaches the MT (97).

Although kinesin stepping simulations have proven useful in understanding motor kinetics, they failed to account for specific states that may be brought about by E-hooks. As such, a kinesin stepping simulation was generated using a Gillespie algorithm, which models a reaction based on known steps and rates (98). Three new stages of the cycle were proposed: accelerated dissociation of ADP-bound kinesin, which would increase velocity and processivity (71,99), additional attraction from E-hooks to allow successful capture of kinesin to the MT (96), and E-hooks as nets that catch kinesins to prevent premature dissociation. A coarse-grained MD framework incorporating this algorithm tested each proposed state individually, with reaction rate parameters tuned according to experimental data from the studies that introduced each stage. Although enhanced ADP release partially matched experimental trends, the combination of E-hook attraction and netting resulted in more accurate agreement with experimental data, providing strong evidence for their inclusion within future kinesin stepping simulations (100).

## Outlook and future directions for E-hook research

### Advancing computational parametrization

As E-hook research has advanced, protocols such as those used to connect modeled E-hooks to crystal MT core structures have become standardized; however, a dedicated force field specifically optimized for E-hook simulations has yet to be developed. When parameterizing MD simulations, for example, most studies utilize either a CHARMM or AMBER force field (101,102); however, these inputs are designed to be the middle ground case, attempting to accommodate both ordered and disordered portions of proteins at the same time. There are additional equations and steps that can be taken during parameterization to enhance a force field’s ability to account for the differences of intrinsically disordered proteins, namely expanding the backbone dihedral energy correction and added levels of detail in water-protein interactions due to the unique effect that they have on intrinsically disordered proteins (103).

It has also been discovered that hydrophilic polymers, like MTs, can form an exclusion zone of water near their surface, essentially creating a shield of negative electrostatic potential (104). The polarization layer formed and consequent electrostatic field have been corroborated in both experimental and computational settings with inclusion in parameterization having a significant effect (104,105,106); however, other studies have debated its existence due to conflicting experimental results (107,108). Such polarization effects may underlie the long-range signaling found in several E-hook binding studies and could account for the reduced diffusivity reported upon E-hook removal (109).

### Posttranslational modifications and structural regulation

PTMs have been experimentally shown to significantly influence both the ability of MAPs to bind to MTs and the functional outcomes of those interactions (97,110,111,112,113,114,115). For instance, polyglutamylation can enhance the activity of severases such as katanin and spastin (114,115) while reducing the run length of kinesin-1 (21). Despite these documented experimental effects, the structural consequences of PTMs remain poorly understood. The increased complexity introduced by PTMs through added charge, steric hindrance, and altered flexibility has limited their incorporation into computational models. Expanding simulations to explicitly include PTM-modified E-hooks would be a major step forward in deciphering how these chemical and structural modifications regulate MAP binding and activity. Such efforts would aid in revealing the structural logic underlying the tubulin code and enable predictive, mechanistic models for how E-hook modifications tune MAP behavior.

### Expanding studies of MAP-E-hook interactions

Kinesins other than the widely studied kinesin-1 and kinesin-3 are also affected by E-hooks and their PTMs. Mitotic kinesins such as kinesin-12 and kinesin-14 have been shown to be reliant upon E-hook presence to properly bind to MTs and facilitate directional sliding within the mitotic spindle (116,117). Depolymerization kinesins such as kinesin-8 also cannot function without E-hooks (30). These nonconventional kinesins differ from kinesin-1 and kinesin-3 in their overall structure, reduced processivity, and specialized functions, and they have not garnered the same computational attention. In addition, there are few studies that probe E-hook-dynein interactions, especially regarding the influence of different E-hook isotypes and PTMs on binding and subsequent function (97,118). As such, additional computational studies exploring how E-hooks influence binding dynamics and stepping cycles in these motors could provide useful insight into how E-hook structure drives class-specific motor behaviors.

Many nonmotor MAPs also depend on E-hooks to be functional. However, most MAPs have been minimally studied computationally with respect to their interactions with E-hooks. Such investigation has the potential to reveal contact points, binding conformations, and the mechanisms by which E-hook isoforms regulate a diverse set of MAPs. The following examples highlight the functional diversity of MAPs whose molecular interactions with E-hooks remain largely unexplored through computation.

Tau is an intrinsically disordered MAP expressed in neurons that acts as a MT stabilizer and dynamic instability regulator (119). Tau’s binding to MTs is strongly influenced by E-hooks (19,120). Biochemical studies using subtilisin-digested MTs suggest that α-tubulin is the primary binding partner of tau as removal of α-tubulin E-hooks reduces tau binding to below detectable levels (121). In contrast, other methodologies have painted different pictures about tau interactions. MD simulations indicate a significant β-tubulin binding preference, potentially due to specific β-tubulin E-hook isotypes (22). Cryo-EM studies further suggest that tau interacts with both the globular core of β-tubulin and the E-hook of α-tubulin (122,123). Notably, tau’s role in regulating dynamic instability overlaps with the proposed function of α-tubulin E-hooks, suggesting the possibility of similar regulatory mechanisms. Computational modeling of E-hook-tau interactions revealed E-hook variability and mobility in bound conformations that contributed to continuing stabilization and provided binding competition for motor proteins (124). Although many studies have determined key properties and functions of tau, further experimental and computational work, combined with cross-validation, is needed to reconcile discrepancies across the literature.

Other functionally diverse MAPs that rely on E-hooks are involved in mitosis and MT severing. hINO90 is a complex responsible for DNA repair, replication, and stability (125,126,127), but during mitosis, it also assists in MT spindle formation (128). Failure to assist in this role leads to spindle defects and errors in chromosome segregation (126). hINO80 contains a lysine-rich K-loop, suggesting that E-hooks serve as the primary site (31). Supporting this, subtilisin treatment of MTs resulted in no hINO80 binding, highlighting the pivotal role of E-hooks for both hINO80 and proper mitosis (128). Severases, such as katanin and spastin, cut polymerized MTs and modulate MT dynamics. Both require E-hooks to bind and properly sever MTs, with their activity significantly regulated by PTMs (115,129,130,131,132). Experimental studies determined key binding domains necessary for severing function (130,131); however, their specific mechanisms remained elusive. It was proposed that the active oligomers of these severing enzymes engaged the acidic E-hooks to initiate their activity, and glutamylation improved severase activity (115). Mutations in severases or disruptions in E-hook regulation can lead to neurodegenerative or muscular disorders (133,134), underscoring the delicate balance of MT dynamic instability. Computational simulations could provide further insight into the mechanisms underlying severase reliance on E-hooks and how subtle perturbations in these interactions result in severe functional consequences.

### Single-isoform E-hook systems

The natural heterogeneity of E-hooks in commonly used MT sources has historically made it difficult to obtain experimental data specific to a single E-hook isoform. As a result, most studies have approached E-hooks on an “all-or-nothing” basis, where MTs either retain all E-hooks or lack them entirely. Investigations of individual E-hook isoforms have therefore relied heavily on computational approaches, highlighting the important role simulations play in this area. A major advance came with the development of a method to produce recombinantly expressed, single-isoform human tubulin (135). In this approach, custom genes for TUBA1 and TUBB3 were synthesized, each containing a distinct tag, and cloned into a dual vector system. These constructs were expressed in cell culture and purified from lysates using chromatography. This methodology now enables more precise experimental exploration of E-hook functions within MT systems. Moreover, it provides key validation for computational studies, allowing experimentally determined parameters, such as dissociation constants, to supplement or refine predictions that were previously predicted from modeling.

## Conclusion

Recent experimental and computational studies have begun to reveal how E-hook isotypes and their PTMs influence MAP binding and function. Computational analyses of kinesin/E-hook interactions suggest new steps in the kinesin mechanochemical cycle, whereas structural modeling highlights functional differences between α- and β-tubulin E-hooks. Simulations indicate that increased E-hook disorder can stabilize complexes and enhance MAP mobility. Together, these findings clarify how subtle compositional changes in E-hooks translate to specific conformational effects and higher-order interactions, providing insight into the mechanisms of the tubulin code and the molecular basis of tubulopathies.

## Data and code availability

This study is a review and did not generate any new data. All data and findings summarized here are available in the original publications cited in the references.

## Acknowledgments

This work was supported by 10.13039/100000002NIH R35GM147030.

## Author contributions

All authors contributed to the conceptualization, literature review, and writing of the manuscript. A.C.B. organized and drafted the manuscript and figures. A.C.B. and D.N.R. contributed to revisions and refinement of the conceptual framework. A.C.B. and D.N.R. wrote, reviewed, edited, and approved the final version of the manuscript.

## Declaration of interests

There are no conflicts of interest to declare.
